# A Narrative Review on Plant Extracts for Metabolic Syndrome: Efficacy, Safety, and Technological Advances

**DOI:** 10.3390/nu17050877

**Published:** 2025-02-28

**Authors:** Hammad Ullah, Marco Dacrema, Daniele Giuseppe Buccato, Marwa A. A. Fayed, Lorenza Francesca De Lellis, Maria Vittoria Morone, Alessandro Di Minno, Alessandra Baldi, Maria Daglia

**Affiliations:** 1 Department of Pharmacy, University of Napoli Federico II, Via D. Montesano 49, 80131 Naples, Italy; marcodacrema1991@gmail.com (M.D.); d.buccato@gmail.com (D.G.B.); lo.delellis2@gmail.com (L.F.D.L.); alessandro.diminno@unina.it (A.D.M.); alessandra.baldi.alimenti@gmail.com (A.B.); 2 School of Medicine, Xi’an International University, Xi’an 710077, China; 3 Department of Pharmacognosy, Faculty of Pharmacy, University of Sadat City, Sadat 32897, Egypt; marwa.fayed@fop.usc.edu.eg; 4 Department of Experimental Medicine, Section of Microbiology and Clinical Microbiology, University of Campania “L. Vanvitelli”, 80138 Naples, Italy; mariavittoria.morone@unicampania.it; 5 CEINGE-Biotecnologie Avanzate, Via Gaetano Salvatore 486, 80145 Naples, Italy; 6 International Research Center for Food Nutrition and Safety, Jiangsu University, Zhenjiang 212013, China

**Keywords:** plant extracts, functional foods, metabolic syndrome, efficacy, safety, technological advancements

## Abstract

Metabolic syndrome, a global health concern, is characterized by visceral obesity, hyperglycemia, dyslipidemia, hypertension, and chronic low-grade inflammation. Current therapeutic options are limited by their varying efficacy and significantly adverse side effects, fueling interest in natural products, particularly plant extracts, as potential preventive interventions for high-risk individuals. This review examines the role of plant extracts in mitigating metabolic syndrome risk factors, addressing safety concerns and exploring associated technological advancements. The literature indicates that plant extracts hold promise for addressing the pathophysiology of metabolic dysfunction. However, challenges such as safety concerns, a lack of standardized regulation, and potential drug–plant interactions currently limit their clinical application. Rigorous, long-term clinical trials are necessary to confirm the efficacy and safety of plant extracts before they can be established as a preventive strategy for managing metabolic syndrome.

## 1. Introduction

Metabolic syndrome is a complex cluster of conditions, including obesity, hyperglycemia, hypertension, dyslipidemia, and a chronic pro-inflammatory state [1,2]. These factors significantly increase the risk of cardiovascular disease, type 2 diabetes mellitus (T2DM), and cerebrovascular accidents. Also known as Reaven’s syndrome, Syndrome X, the deadly quartet, or insulin resistance syndrome, its precise definition and diagnostic criteria vary across organizations like the World Health Organization (WHO), the National Cholesterol Education Program’s Adult Treatment Panel III (NCEP ATP III), and the International Diabetes Federation (IDF) [3,4,5]. However, all definitions emphasize the importance of visceral obesity, dyslipidemia, hypertension, and hyperglycemia.

The prevalence of metabolic syndrome has risen globally in recent decades, particularly among urban populations in developing countries [6]. A recent meta-analysis of data from 28 million individuals estimated a global adult prevalence of 12.5–31.4%, depending on diagnostic criteria [7], with significantly higher rates observed in the Eastern Mediterranean Region and the Americas. Prevalence rates for individual components are as follows: 45.1% for ethnicity-specific central obesity, 42.6% for elevated blood pressure (systolic ≥130 mmHg and/or diastolic ≥85 mmHg), 40.2% for reduced HDL-cholesterol (<1.03 mmol/L for men or <1.29 mmol/L for women), 28.9% for elevated triglycerides (≥1.7 mmol/L), and 24.5% for elevated fasting plasma glucose (≥5.6 mmol/L) [7]. The WHO has also reported alarmingly high global figures for obesity (890 million), diabetes (422 million), and hypertension (1.28 billion) [8,9,10].

Risk factors for developing metabolic syndrome include female sex, an age of over 50 years, a sedentary lifestyle, a family history of metabolic syndrome, low socioeconomic status, illiteracy, unemployment, an omnivore diet, stress, insomnia, and a high body mass index (BMI) [11]. Untreated or poorly managed metabolic syndrome can lead to serious health complications, including coronary heart disease (CHD), heart failure, stroke, hepatic steatosis, and liver failure, presenting a major challenge to global healthcare systems [12,13]. Current management strategies primarily involve lifestyle modifications (aerobic exercise and dietary changes) and pharmacological interventions targeting individual components of the syndrome [14,15,16]. However, the numerous and potentially severe adverse drug reactions (ADRs) associated with these treatments, including lactic acidosis (metformin), congestive heart failure (thiazolidinediones), renal toxicities and hyperkalemia (RAAS inhibitors), peptic ulcers (aspirin), and myopathies (hypolipidemics), often outweigh the benefits [17,18,19,20].

Natural products from marine and terrestrial sources offer significant potential for promoting human health and managing challenging diseases [21]. The growing global market for natural substances reflects a rising consumer preference for preventative healthcare [22]. Indeed, a 2014 report indicated that over 80% of the world’s population utilizes botanical products for primary healthcare [23]. Herbal products have demonstrated efficacy in randomized controlled trials for reducing metabolic syndrome risk factors by positively influencing blood pressure, serum glucose levels, waist circumference, and lipid levels [24], suggesting that they may provide valuable alternative treatment options.

This review examines the efficacy and safety of plant extracts in mitigating metabolic syndrome risk factors. We consider the challenges and opportunities presented by current extraction and encapsulation techniques and aim to achieve the following: (i) assess the evidence on the efficacy of plant extracts in managing metabolic syndrome risk factors; (ii) evaluate their safety profiles based on existing data; and (iii) assess technological advancements in extract preparation and delivery methods relevant to clinical applications. This review synthesizes information from 139 articles identified through a comprehensive literature search in PubMed, Scopus, Web of Science, and the Cochrane Library. Plant extracts were included based on their documented efficacy in managing metabolic disorders. Articles focusing on extracts with limited or inconclusive metabolic effects were excluded. Additionally, studies addressing safety, bioavailability, or technological advancements related to plant extracts were prioritized.

## 2. Plant Extracts as Functional Food Ingredients

**Figure 1:**
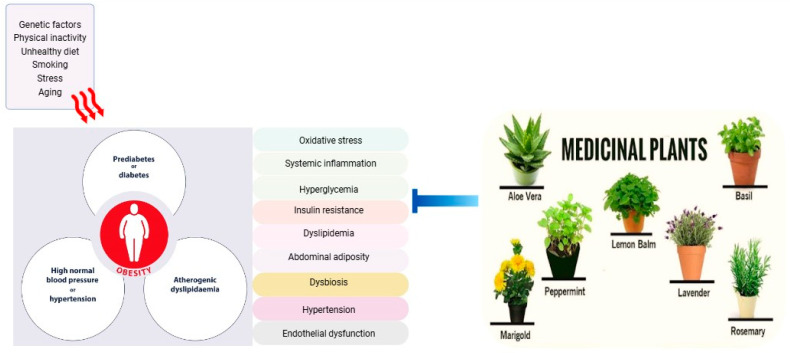
Factors contributing to metabolic syndrome and potential targets of medicinal plants in mitigating risk factors.

A diverse range of fruits and vegetables are rich in bioactive compounds such as polyphenols (flavonoids, phenolic acids, and stilbenes), carotenoids, organosulfur compounds, and dietary fibers, which offers a promising approach to mitigating metabolic syndrome risk factors. Polyphenols and carotenoids can reduce oxidative stress, improve insulin sensitivity, and enhance glucose metabolism. Organosulfur compounds may improve glucose and lipid metabolism and reduce systemic inflammation, while dietary fibers are best known for their role in regulating gut microbiota, enhancing satiety, and improving insulin sensitivity [25,26]. These foods demonstrate potential benefits in improving glucose and lipid homeostasis, reducing ectopic lipid deposition, lowering inflammatory markers, and modulating the gut microbiome. Furthermore, bioactive compounds within these foods can modulate multiple signaling pathways, affecting enzyme activity, gene expression, epigenetic regulation, and protein expression [27]. The incorporation of plant extracts and their bioactive components into functional foods and supplements presents a novel strategy for preventing metabolic syndrome, particularly among high-risk individuals. Figure 1 illustrates the factors contributing to metabolic syndrome and the potential targets of medicinal plants in mitigating its risk factors.

### 2.1. Obesity and Hyperglycemia

Obesity is characterized by excessive body fat accumulation and is accompanied by adverse alterations in adipose tissue, including reduced lipid turnover and increased infiltration of inflammatory macrophages [28]. Visceral and abdominal adipose tissue, in particular, negatively impacts metabolic and insulin signaling pathways, contributing to the pathogenesis of obesity and other metabolic syndrome risk factors [29]. Chronic hyperglycemia, resulting from impaired insulin secretion and/or action, can progress silently to T2DM, leading to potentially severe complications including retinopathy, neuropathy, nephropathy, atherosclerosis, peripheral arterial disease, and cerebrovascular accidents [30,31,32]. In most cases, both insulin resistance and impaired insulin secretion contribute to the metabolic disturbances affecting carbohydrate, protein, and lipid metabolism.

Several studies highlight the potential of specific plant extracts in addressing obesity and hyperglycemia. A study by Ullah et al. [33] demonstrated that a polyphenol-rich hydroethanolic extract of *Prunus domestica* L. inhibited key enzymes involved in glucose and lipid metabolism (α-amylase, α-glucosidase, HMG-CoA reductase, and pancreatic lipase) in vitro. Supplementation with *Prunus persica* (L.) Stokes flower extract (0.2% or 0.6%) for eight weeks significantly reduced body weight, visceral fat mass, and serum levels of glucose, alanine aminotransferase (ALT), and aspartate aminotransferase (AST) in a high-fat, diet-induced, obesity mouse model [34]. These effects were linked to improved hepatic lipid metabolism. *Berberis* species, rich in berberine, have demonstrated anti-diabetic effects in in vitro, in vivo, and clinical studies [35]. Berberine’s mechanism involves promoting glucose uptake, inhibiting gluconeogenesis through SIRT3 inhibition, and mimicking insulin-sensitizing effects via protein tyrosine 1B downregulation [36,37,38,39]. *Hibiscus sabdariffa* L., *Vigna unguiculata* L. Walp., and *Solanum nigrum* L. extracts significantly reduced fasting blood glucose levels in a high-fat, diet-streptozotocin-induced, diabetic rat model [40].

A randomized, placebo-controlled, crossover trial showed that consuming Queen Garnet plum juice for 28 days significantly decreased body weight, BMI, and leptin levels, and increased adiponectin levels in healthy participants [41]. Another randomized, placebo-controlled, crossover trial demonstrated that soluble fiber-rich brewer’s spent grain significantly reduced postprandial glycemia and insulinemia in healthy individuals with slightly impaired glucose tolerance [42]. The extensive literature has reported the pronounced health benefits of a Mediterranean diet rich in olive oil (containing monounsaturated fatty acids and polyphenols), particularly on metabolic health [43,44]. A randomized clinical trial showed an attenuation of the early postprandial glycemic response in type 1 diabetic patients who consumed extra-virgin olive oil mixed with a high-glycemic index meal [45]. Another study demonstrated a significant reduction in fasting blood glucose, total cholesterol (TC), low-density lipoprotein (LDL), and triglycerides in diabetic subjects who consumed olive oil (30 mL/day) for four weeks [46]. In addition, high-density lipoprotein (HDL) levels were considerably increased with olive oil consumption. The oral administration of olive leaf extract (500 mg/day) for 14 weeks significantly decreased HbA1c and fasting insulin levels in diabetic subjects in a randomized, placebo-controlled trial [47].

Additional studies using fenugreek seeds [48,49,50] and green tea extract [51,52,53] are also described. Gupta et al. [48] observed significant improvements in the area under the curve of glucose and insulin sensitivity in diabetic patients supplemented either with fenugreek seeds (1 g/day) or a placebo for two months, though no significant differences were noted in fasting blood glucose levels or an oral glucose tolerance test among these groups. Another study with a cross-over design showed a considerable decrease in fasting blood glucose levels and an improvement in an oral glucose tolerance test in diabetic subjects supplemented with a diet containing fenugreek seeds (100 g/day) for 10 days [49]. The supplementation of diabetic subjects with fenugreek seeds (15 g/day) soaked in water resulted in a significant reduction in postprandial glucose levels [50]. Green tea, one of the most common beverages in the world used for maintaining a normal body weight and glucose metabolism, showed controversial results in clinical trials. A randomized, placebo-controlled study exhibited a significant decrease in HbA1c levels with no considerable effects on the fasting blood glucose levels of healthy subjects who consumed a packet of green tea extract (containing 544 mg polyphenols) daily for two months [51]. A combination of exercise and green tea extract (containing 890 mg polyphenols) resulted in a significant decrease in the area under the curve for insulin, with an increase in insulin sensitivity in healthy subjects [52]. Conversely, another study showed no effects on fasting blood glucose and HbA1c levels, insulin sensitivity and secretion, and glucose tolerance in healthy subjects supplemented with epigallo-catechin-3-gallate (800 mg/day) for eight weeks [53]. A study by Wang et al. [54] compared the beneficial effects of Qinggan Jiangtang and Glucophage tablets in patients with metabolic syndrome through a randomized, controlled, double-blind clinical trial. The results demonstrated that both treatments significantly reduced blood glucose levels, lipid profiles, blood pressure, and insulin resistance. However, no statistically significant differences were observed between the two interventions in terms of their overall efficacy. While assessing the potential benefits of grape seed extract (rich in resveratrol and procyanidins) against insulin resistance in Iranian adolescents with metabolic syndrome, Mohammed et al. [55] observed significant improvements in insulin concentration and insulin resistance after supplementing participants with grape seed extract (100 mg/day) for eight weeks.

### 2.2. Dyslipidemia

Dyslipidemia, a key risk factor for atherosclerosis and subsequent cardiovascular events, is characterized by abnormal lipid profiles: elevated levels of TC, LDL, very-low-density lipoprotein (VLDL), and triglycerides (TGs), coupled with low levels of high-density lipoprotein (HDL) [54,55]. Dyslipidemia is strongly associated with obesity and/or T2DM [56,57], and risk factors include smoking, excessive alcohol consumption, obesity, T2DM, and certain medications (e.g., steroids) [58]. The Mediterranean diet, rich in fruits, vegetables, legumes, complex carbohydrates, unsaturated fatty acids, moderate wine consumption, and fish, while limiting red meat and dairy, has demonstrated significant protective effects against dyslipidemia [59,60].

Numerous studies have investigated the effects of various plant extracts on dyslipidemia. Extracts from the fruits, leaves, and bark of *Zanthoxylum armatum* DC (500 mg/kg) demonstrated significant hypolipidemic effects in mice treated for 15 days. A reduction in TC, TG, and LDL levels was observed [61]. A novel herbal formula (Schisandrae Fructus, milk thistle, hawthorn, and bitter melon) demonstrated efficacy in ameliorating diet-induced metabolic syndrome [62]. In vitro studies indicated the potent inhibitory effects of the formula’s components on adipocyte differentiation, cholesterol uptake, and hepatic lipid accumulation. In vivo studies demonstrated reduced body weight, fat pad mass, and liver weight, and improved lipid profiles. Fixed oils from spices (*Alpinia galanga* (L.) Willd., *Cinnamomum zeylanicum* var. cassia, *Trigonella foenum-graecum* L., *Foeniculum vulgare* Mill., and *Myristica fragrans* Houtt.) showed an in vitro reduction in accumulated lipid droplets in 3T3-L1 cell lines, and an in vivo improvement of lipid profiles, anti-oxidant enzymes, and reduced droplets in liver and adipose tissues in C57BL/6 mice [63]. Another study showed a significant decrease in TC, LDL, TG, and the atherogenic index, and an increase in HDL levels in diet-induced dyslipidemia in Wistar rats treated with *Mangifera indica* L. leaf extract [64]. An interventional study by Venturini et al. [65] demonstrated a significant decrease in oxidative capacity and an improvement in cholesterol parameters (TC, LDL, and HDL) in subjects with metabolic syndrome, co-supplemented with fish oil (3 g/day) and extra-virgin olive oil (10 mL/day) for three months.

De Lellis et al. [66] observed the hypolipidemic effects of food supplements based on monacolins, γ-oryzanol, and γ-aminobutyric acid (bioactive ingredients from rice fermented with the *Monascus purpureus*) in participants with mild dyslipidemia. In a randomized, double-blind, placebo-controlled trial, enrolled subjects were treated either with a supplement or a placebo for three months, and the results indicated a significant decrease in TC and LDL and an increase in HDL levels in the supplement-treated group. The daily consumption of prunes (100 g) for eight weeks resulted in a significant reduction in serum LDL levels and fecal bile concentration of lithocholic acid as compared to grape juice (control) in a cross-over study [67]. A randomized clinical study demonstrated a considerable improvement of TC, TG, LDL, and HDL in hyperlipidemic subjects treated with lettuce seed extract (1000 mg/day) for 12 weeks [68]. Eight weeks of intake of a nutraceutical supplement based on bergamot extract (120 mg flavonoids), vitamin C, phytosterols, and chlorogenic acid from dry artichoke extract significantly improved the levels of TC, TG, LDL, non-HDL cholesterol, high sensitivity C-reactive protein (hs-CRP), and tumor necrosis factor-alpha (TNF-α) in a three-arm, placebo-controlled trial in dyslipidemic, overweight subjects [69]. A three-times daily intake of bitter melon extract (100 mg) for 30 days significantly reduced LDL levels as compared to a placebo in Japanese adults, though no significant difference was observed among the groups in TC, TG, and blood glucose levels [70]. A participant-blinded, randomized, placebo-controlled, crossover trial showed a significant improvement in fat distribution and lipid profiles in healthy adolescents supplemented with psyllium fibers (6 g/day) for six weeks [71].

### 2.3. Hypertension, Endothelial Dysfunction, and Pro-Inflammatory State

The etiology of hypertension in metabolic syndrome is multifactorial, involving insulin resistance, obesity, hyperglycemia, and dyslipidemia [72,73,74]. Hypertension is a major risk factor for cardiovascular and cerebrovascular complications. A substantial body of evidence supports dietary modifications as effective strategies for preventing or managing hypertension [75]. Diets rich in fruits, vegetables, whole grains, and low-fat dairy products, while minimizing sodium intake, are particularly beneficial. Furthermore, reducing inflammation and improving endothelial function are crucial components of metabolic syndrome therapy in order to prevent or delay the onset of chronic complications [76].

Polyphenol-rich diets (e.g., those including tea, red wine, fruits, and vegetables) modulate vascular tone by upregulating the nitric oxide–cyclic guanosine monophosphate (NO-cGMP) pathway, and they mitigate oxidative stress by reducing the production of endogenous reactive oxygen species (ROS) such as NADPH oxidase [25]. Luna-Vazquez et al. [77] demonstrated that a chemically characterized black cherry fruit extract (300 mg/kg/day), rich in polyphenols (chlorogenic acid and anthocyanins), significantly reduced oxidative stress markers and systolic blood pressure in an L-NAME-induced, hypertensive rat model. Similarly, treatment with a methanolic extract of *Adansonia digitata* L. (200 mg/kg and 400 mg/kg/day) dose-dependently reduced systolic and diastolic blood pressure, mean arterial pressure, and heart rate to normal physiological levels [78]. Furthermore, *A. digitata* extract reduced biomarkers associated with endothelial dysfunction (angiotensin-converting enzyme activity), inflammation (C-reactive protein and IL-1β), oxidative stress (malondialdehyde), and cardiac injury (creatine kinase-MB and lactate dehydrogenase).

Kim et al. [79] studied the vasorelaxant effects of *Prunus persica* extract on endothelium-denuded aortic rings from a rat thoracic aorta using concentrations ranging from 0.5 to 20 μg/mL. They found that the extract’s vasorelaxation involved the nitric oxide–soluble guanylate cyclase–cyclic guanosine monophosphate (NO-sGC-cGMP) pathway, vascular prostacyclin, and muscarinic receptor transduction. Additionally, the extract reduced calcium-induced vasoconstriction via inositol triphosphate receptors (IP3Rs) in the endoplasmic reticulum membrane. A randomized, controlled, cross-over clinical trial showed a significant reduction in systolic blood pressure in adults with mildly elevated blood pressure who consumed cruciferous vegetables (300 g/day) as compared to root and squash vegetables [80]. The supplementation of subjects with mild hypertension with *Nigella sativa* L. seed extracts (200 and 400 mg per day) for eight weeks resulted in a significant reduction in systolic and diastolic blood pressure in a randomized, placebo-controlled clinical trial [81]. The consumption of garlic may enhance nitric oxide production, improve endothelial function, and reduce oxidative stress, thereby improving blood pressure [82]. A double-blind, randomized, placebo-controlled clinical trial showed that the supplementation of hypertensive subjects with aged garlic extract (960 mg/day, containing 2.4 mg S-allylcysteine) for 12 weeks reduced systolic blood pressure in treated patients with uncontrolled hypertension [83].

Supplementation with anthocyanin-rich Queen Garnet plum juice alleviates platelet aggregation via reduced P-selectin expression of activated de-granulated platelets, increased activated partial thromboplastin clotting time, and decreased blood levels of fibrinogen and malondialdehyde in healthy volunteers in a randomized, placebo-controlled clinical trial [84,85]. An experimental study designed to verify the potential of dietary berries (*Viburnum trilobum* Marshall, *Amelanchier alnifolia*, *Shepherdia argentea* (Pursh) Nutt., and *Prunus virginiana* L.) in alleviating diabetic microvascular complications and pro-inflammatory gene expression showed the potent inhibition of aldose reductase with a nonpolar fraction (rich in carotenoids) and the strong inhibition of IL-1β and COX-2 gene expression with polar fraction (rich in anthocyanins, phenolic acids, and proanthocyanidins) [86]. The aldose reductase enzyme is reportedly involved in the pathogenesis of diabetic microvascular complications.

The potential effects of plant extracts on metabolic syndrome risk factors from in vitro, in vivo, and clinical studies are summarized in Table 1.

## 3. Safety Concerns

The safety of plant extracts and their bioactive constituents is paramount when considering their use in dietary supplements or functional foods. Although generally considered safe, thorough evaluation is crucial to ensure human safety, as many plant extracts lack systematic toxicity testing [87]. A significant number of plants used traditionally as food or medicine have demonstrated potential toxicity, mutagenicity, or carcinogenicity [87]. Adewunmi and Ojewole [88] identified several potentially toxic compounds found in complementary and alternative medicines: lectins, viscotoxins, aristolochic acids, pyrrolizidine alkaloids, benzophenanthrine alkaloids, saponins, diterpenes, cyanogenic glycosides, and furanocoumarins.

Unlike conventional pharmaceuticals, dose-dependent toxicity data and long-term safety evaluations for plant extracts are often limited, especially for novel or high-potency formulations [89]. Several commonly used plant extracts, for example, *Curcuma longa* L., *Camellia sinensis* (L.) Kuntze, *Withania somnifera* (L.) Dunal, *Garcinia gummi-gutta* (L.) N. Robson, *Monascus purpureus*, and *Actaea racemosa* L., have been associated with increased hepatotoxicity risk [89].

Additional safety concerns arise from microbial or heavy metal contamination during the harvesting, processing, and storage of plant materials [90]. Heavy metal contamination, resulting from various industrial, agricultural, and technological sources, is often found in herbal products at concentrations exceeding permitted limits [91]. These heavy metals are known carcinogens and can cause internal organ toxicity affecting the brain, heart, lungs, liver, and kidneys [91]. The WHO emphasizes the need for international standards and procedures for assessing the safety and efficacy of traditional medicines [92]. Although pharmacological and toxicological data are crucial for drug development, such data for plant extracts are far less abundant than reports of their purported therapeutic benefits [93,94,95,96]. Rigorous in vivo safety and efficacy studies, using appropriate animal models and well-designed, randomized, placebo-controlled clinical trials, are essential [92,97]. Comprehensive long-term safety evaluations are critical for the development of standardized herbal medicines and their adoption by healthcare providers.

Finally, drug–botanical interactions pose a growing concern [98]. These interactions may be pharmacokinetic (affecting drug absorption, distribution, metabolism, and elimination) or pharmacodynamic (producing antagonistic, synergistic, or additive effects). Grapefruit products, for instance, inhibit intestinal cytochrome P450 3A4 (due to furanocoumarins) and modulate P-glycoprotein and drug transporters (due to flavonoids), altering the bioavailability of many drugs, including calcium channel blockers, statins, antihistamines, and immunosuppressants [99]. American ginseng and cranberry juice can affect warfarin metabolism, impacting coagulation and bleeding risk [100,101]. Similarly, ginkgo products can interfere with P-glycoprotein-mediated drug transport, reducing blood and tissue concentrations of several drugs, such as colchicine, doxorubicin, digoxin, quinidine, tacrolimus, verapamil, and rosuvastatin [98].

Regulations governing the use of plant extracts for managing risk factors associated with metabolic syndrome vary significantly across countries. In the United States, plant extracts are regulated by the Food and Drug Administration (FDA) under the Dietary Supplement Health and Education Act (DSHEA). This framework ensures that these products are safe for consumption and are accurately labeled. However, unlike pharmaceutical drugs, plant extracts do not require pre-market approval. In contrast, within the European Union, plant extracts are regulated by the European Medicines Agency (EMA) and are classified either as food supplements or medicinal products, depending on their intended use. Regardless of the regulatory framework, plant extracts intended for the management of metabolic syndrome risk factors must meet stringent efficacy and safety standards to ensure their therapeutic potential and minimize health risks [102].

## 4. Technological Aspects

Plant extracts have a long history of use as preventive and therapeutic agents for managing metabolic syndrome risk factors [103]. Rich in bioactive compounds, these extracts offer promising potential for mitigating obesity, hyperglycemia, hypertension, and dyslipidemia. However, widespread clinical application requires a thorough understanding of the technological aspects of their production, stabilization, and delivery. Recent advancements in extraction and processing techniques have significantly improved the stability, bioavailability, efficacy, and safety of bioactive compounds, enhancing their effectiveness in both supplements and pharmaceutical formulations [104,105].

The technological aspects of plant extract utilization are multifaceted, ranging from selecting optimal extraction methods to developing innovative delivery systems. Emerging technologies, including ultrasound-assisted extraction, supercritical fluid extraction, and encapsulation, offer significant advantages over traditional methods: enhanced efficiency, higher yields, improved compound stability, and reduced environmental impact. This section will explore the technological aspects of plant extract processing, focusing on extraction techniques, delivery systems, stability enhancement strategies, formulation approaches, and industrial-scale production.

### 4.1. Extraction Techniques

**Figure 2:**
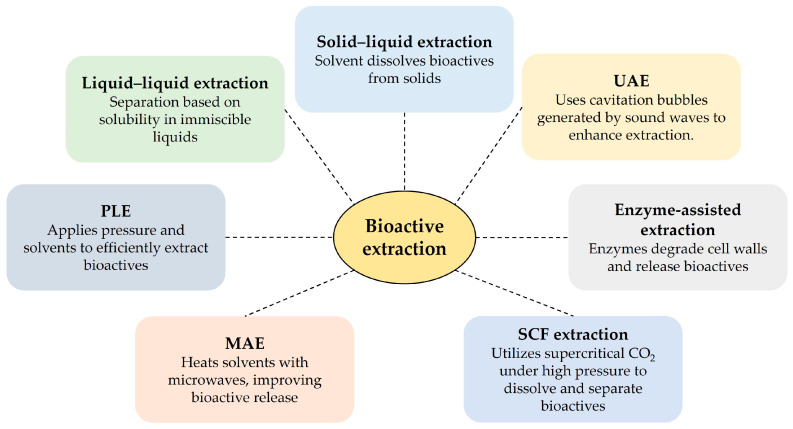
Extraction techniques and their basic principles. Ultrasound-assisted extraction (UAE); microwave-assisted extraction (MAE); pressurized liquid extraction (PLE); and super critical fluid (SCF).

Extraction is the initial step in isolating and purifying bioactive compounds from botanical and food sources. Soluble compounds are generally easier to extract than insoluble secondary metabolites such as flavonoids and phenolic acids. While Soxhlet, maceration, and heat reflux are established methods, their equipment requirements vary. Optimal extraction technologies prioritize product quality, efficiency, cost-effectiveness, and sustainability [104]. The food industry is actively exploring novel extraction methods to meet consumer demand for chemical-free, sustainably produced products. Cutting-edge techniques such as ultrasound-assisted extraction (UAE), enzyme-assisted extraction, microwave-assisted extraction (MAE), pressurized liquid extraction (PLE), and supercritical fluid extraction (SFE) are rapidly replacing traditional methods [106]. These innovative approaches often result in higher yields, improved extraction rates, reduced energy consumption, and better preservation of thermosensitive compounds [106]. Figure 2 summarizes these techniques.

Traditional methods, such as liquid–liquid extraction, solid-phase extraction, and solid-phase microextraction, have also been used. Liquid–liquid extraction utilizes two immiscible solvents (e.g., aqueous and organic solvents) to partition the analyte based on its relative solubility in each solvent [107]. Solid-phase extraction uses a solid stationary phase to selectively adsorb or extract analytes from a liquid sample [108]. Solid-phase microextraction exposes a sample to a solid phase coated with an extracting phase for a defined period, followed by analysis using gas chromatography or high-performance liquid chromatography (HPLC). This method is particularly useful for detecting trace amounts of bioactive compounds [109].

UAE utilizes ultrasonic frequencies (18–100 kHz), inaudible to humans, to enhance mass transfer and disrupt cellular matrices, thereby increasing extraction yields [110,111]. Enzyme-assisted extraction employs enzymes to break down cell walls, improving solvent access to bioactive compounds [112]. MAE uses microwave energy for both the internal and external heating of the sample matrix, avoiding thermal gradients and enhancing extraction efficiency [113]. PLE applies pressure to maintain the liquid state of the solvent at elevated temperatures (50–200 °C), increasing extraction efficiency [114]. SFE utilizes supercritical fluids (e.g., CO2) whose properties can be finely tuned by adjusting the temperature and pressure to achieve optimal selectivity and efficiency [115].

Using ultrasound energy to extract tea solids from dried leaves with water increased the extraction yield by 20%. Using several solvents, such as ethanol, ethyl acetate, and butanone, UAE also demonstrated superior carnosic acid extraction and decreased extraction time [116]. Jadhav et al. [117] demonstrated the enhanced extraction of vanillin in a shorter time period for different solvents using UAE technique as compared to the Soxhlet method. Cho et al. [118] showed UAE as being very effective method for extracting resveratrol from grapes, where the degradation of resveratrol during extraction process was negligible within a specified time period. When compared to maceration and Soxhlet extraction, UAE provides the maximum extraction yield of some flavonoids, including tectoridin, iristectorin B, iristectorin A, tectorigenin, iris-tectorigenin A, and total isoflavones, in a shorter amount of time [119]. MAE, an alternate method for extracting tanshinones from the root of *Saliva miltiorrhiza* Bunge, yields higher extraction efficiency in less time [120]. A kinetic analysis of the impact of the solvent composition, the solvent volume, the extraction temperature, and matrix properties on the MAE of peppermint and rosemary leaves showed that using pure, microwave-transparent solvents like hexane could lead to the quick extraction of essential oil components from sample matrices that contain water. This resulted from the direct contact of microwaves with the cell’s free water molecules, which ruptured the cell and released the essential oil into the hexane [121]. The MAE-prepared extract had the highest scavenging activity and the highest phenolic and tannin concentration. MAE was found to be more effective than UAE in terms of extraction efficiency, especially when it came to extracting the phenolic and tannin content. Additionally, a notable 20% increase in antioxidant activity was seen [122].

SFE is used to extract volatile or aromatic chemicals from plant materials, including caffeine and essential oils. Several variables are crucial for extraction by SFE, including temperature, pressure, sample volume, cosolvent addition, and flow and pressure control [116]. Hexane, pentane, butane, nitrous oxide, sulfur hexafluoride, and fluorinated hydrocarbons are among the solvents that can be utilized for SFE, with CO_2_ being the most widely used extraction solvent [123]. There are several benefits to using SFE with CO_2_ for grape seed oil extraction in terms of both process efficiency and extracted oil quality. Supercritical CO_2_ extraction produces oil devoid of organic solvents, and it also takes less processing time than traditional solvent extraction techniques. Today’s oil technology requires the extract to be completely free of organic solvents; otherwise, it takes a lot of time and effort [124]. Kothari et al. [125] conducted a comparative analysis of different extraction techniques for extracting phenolic and antibacterial components from plant seeds (*Annona squamosa*, *Manilkara zapota*, *Phoenix sylvestris*, *Syzygium cumini*, and *Tamarindus indica*). These techniques included the Soxhlet method, UAE, extraction by continuous shaking at room temperature, and MAE, both with and without intermittent cooling. The Soxhlet technique was more effective in terms of higher extraction efficiency and phenolic compound extraction. MAE with intermittent cooling, room temperature extraction by shaking, and UAE showed promising effects in extracting antibacterial components from plant seeds.

### 4.2. Encapsulation and Delivery Systems

Despite their wide range of health benefits, the application of plant extracts and their bioactive compounds in functional foods and supplements has been limited by their low bioaccessibility and bioavailability [126,127,128,129,130]. This is often due to several factors, including limited release from the matrix, poor solubility in gastrointestinal fluids, low permeability across epithelial cells, and susceptibility to degradation during gastrointestinal transit [126,127,128,129,130]. Many bioactive compounds are also sensitive to environmental factors such as oxygen and heat, further reducing their effectiveness.

**Figure 3:**
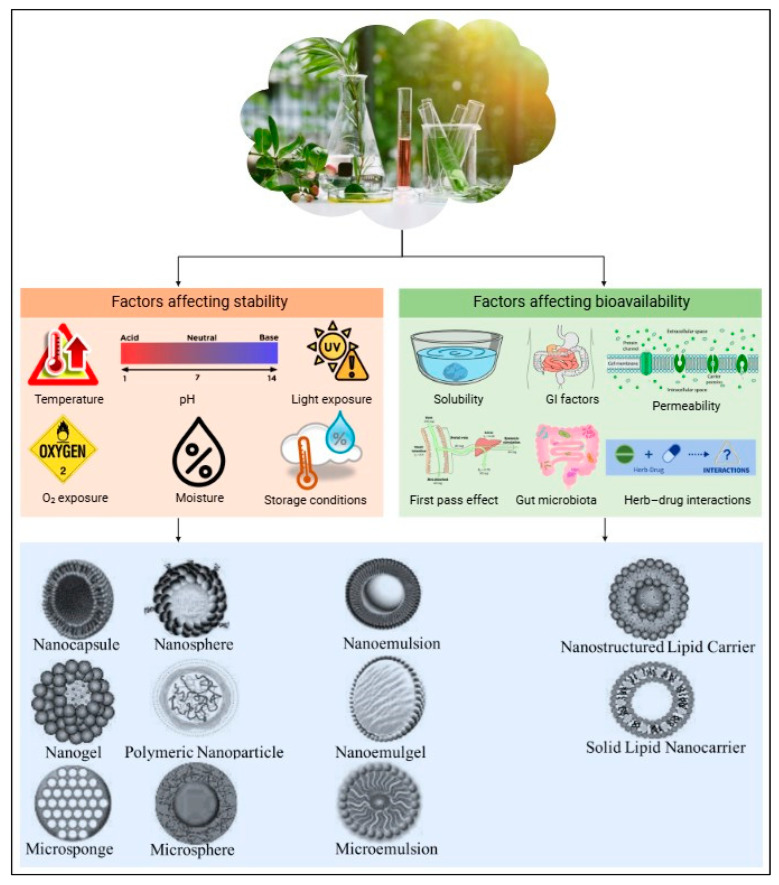
Factors influencing stability and bioavailability of botanical extracts and encapsulation techniques for enhancement.

Encapsulation and advanced delivery systems offer innovative solutions to these challenges, improving the stability and bioavailability of plant extracts [131,132]. Encapsulation involves coating an active compound or mixture with a polymeric material to protect it from environmental degradation and to control the release of the bioactive compounds at specific sites [133]. Encapsulation can also mask unpleasant odors or tastes, improving the overall sensory appeal of the product [134]. Given the often-limited bioavailability of plant-derived bioactives, encapsulation is a promising strategy to protect these compounds during gastrointestinal transit and to enhance their delivery to the target site of action. Figure 3 illustrates various factors that affect the stability and bioavailability of botanical extracts, as well as the different encapsulation techniques used to protect against these factors, thereby improving both stability and bioavailability.

Encapsulation techniques are classified by capsule size as nano-encapsulation (<1 µm) or microencapsulation (3–800 µm) [135,136]. Various methods are currently being explored, including spray drying, freeze drying, extrusion, emulsification, coacervation, molecular inclusion, and ionic gelation (Table 2).

Several encapsulation techniques are employed to enhance the stability and bioavailability of plant extracts. Spray drying, a cost-effective and scalable method, atomizes a mixture of wall material and active ingredients in a hot chamber, causing the solvent to evaporate and the active compound to solidify into a powder [137]. Freeze drying, suitable for temperature-sensitive compounds, freezes the mixture and then removes the ice through sublimation under vacuum, resulting in a porous powder [137]. Extrusion involves forcing a gel solution (often using sodium alginate as the wall material) through a nozzle to create capsules in a hardening bath (e.g., calcium chloride solution) [138]. Emulsification combines two immiscible liquids (e.g., oil and water) stabilized by an emulsifier, producing either a liquid or solid final product [137]. Coacervation involves separating polyelectrolyte phases to encapsulate the active compounds, with cross-linking agents often used to improve stability [136]. Molecular inclusion utilizes cyclodextrins or similar compounds to encapsulate polar molecules through non-covalent interactions [139,140]. Finally, ionic gelation employs biopolymer-based microbeads to encapsulate active compounds, often using calcium alginate [139].

Numerous studies have demonstrated the benefits of encapsulation in improving the delivery and efficacy of plant-derived compounds. Ezzat et al. [141] and Peng et al. [142] reported increased oral bioavailability of encapsulated tea polyphenols in rats. Similarly, coacervated fisetin showed enhanced bioavailability and increased peak plasma concentrations in C57BL/6 mice [143]. Nano-formulated tea extracts demonstrated anti-obesity effects in rats via the modulation of the AMPK/Sirt-1/Glut-4 and PPAR-γ pathways [144]. Freeze-dried mulberry fruit extract improved various metabolic parameters (body weight, adiposity index, glucose intolerance, lipid profiles, atherogenic index, and oxidative stress) in a menopausal, metabolic syndrome animal model [145]. Andean blueberry anthocyanin niosomes reduced fasting blood glucose and insulin levels, glucose intolerance, and body weight [146]. The ionic gelation of black carrot anthocyanin extract reduced lipid peroxidation, increased antioxidant enzyme activity, and decreased lipogenesis [147]. Spray-dried peanut skin extract reduced postprandial glucose spikes [148]. Finally, 14-day toxicity studies in animal models demonstrated the safety of encapsulated extracts from green coffee fruit, polyherbal formulations (PHFs), and *Moringa oleifera* leaf polyphenols [149,150,151]. The encapsulation of cocoa polyphenol extract significantly improved the delivery of flavanols to the gut in a randomized, cross-over clinical trial, thereby enhancing their bioaccessibility and bioavailability [152]. Another study showed increased bioavailability of encapsulated almond skin polyphenols (flavan-3-ols, flavonols, and flavanones) in a single-blind, placebo-controlled, and randomized trial [153]. These studies strongly suggest the safety and potential benefits of using encapsulated plant extracts.

### 4.3. Stabilization and Shelf-Life Improvement

Maintaining the shelf life of plant extracts in functional foods presents a significant challenge. Bioactive compounds, such as phenols and carotenoids, are susceptible to degradation from environmental factors like oxygen, heat, and light, resulting in reduced bioactivity [154,155]. Several technologies, including encapsulation, spray drying, and freeze drying, along with the use of natural stabilizers and antioxidants, are crucial for extending shelf life and maintaining functionality [154,155].

For example, microencapsulation via extrusion extends the shelf life of oxidation-sensitive flavor compounds, such as citrus oils, by creating a nearly impermeable barrier against oxygen diffusion through the hydrophilic glassy matrix [156]. Extruded citrus oils can remain stable for up to five years, compared to one year for spray-dried oils and only a few months for unencapsulated oils [156]. The spray drying of *Euterpe oleracea* Mart. powder reduced moisture content and improved stability, inhibiting microbial growth and chemical degradation [157]. Similarly, the spray drying of grape skin phenolic extracts decreased moisture content and water activity, while freeze drying reduced hygroscopicity [158].

### 4.4. Formulation into Functional Foods, Scalability, and Industrial Applications

The incorporation of plant extracts into functional foods has gained significant attention as a preventative strategy to combat the rising prevalence of metabolic disorders and their associated health consequences. However, several challenges exist. The characteristics of plant extracts may change as production scales up, potentially affecting the flavor, texture, and nutritional value of the final product [159]. Moreover, the inherent instability of many bioactive compounds leads to degradation during processing and storage, reducing efficacy and health benefits [160].

Meeting the growing demand for functional foods necessitates scaling up production, a complex process requiring careful planning and execution. Simple quantity increases are insufficient; several factors change as production scales from small to large volumes. Maintaining desired effects require the careful consideration of bioactive compounds’ interactions within the food matrix, as their behavior can differ significantly at higher concentrations [161]. For example, bioactives such as polyphenols, carotenoids, phytosterols, and peptides, while enhancing nutritional value, may lose bioactivity during processing and storage [161]. Changes in taste and stability can also arise, potentially creating incompatibilities with existing food manufacturing processes. Challenges include adapting sourcing and processing, modifying formulations, and addressing potential operational complications [159,161]. Encapsulation techniques, such as spray drying, freeze drying, and coacervation, are commonly used to overcome these challenges and effectively incorporate bioactive compounds into functional foods [162,163].

## 5. Conclusions

This review underscores the considerable potential of plant extracts in mitigating risk factors associated with metabolic syndrome. Current evidence highlights their ability to regulate glucose and lipid metabolism, improve vascular function, and counteract oxidative stress. Integrating plant extracts into functional foods and supplements presents a promising strategy for enhancing the health-promoting properties of these products and potentially preventing chronic diseases. However, several limitations exist within the body of research investigating the beneficial effects of plant extracts on metabolic syndrome, revealing critical gaps in the literature:

- Limited clinical translation: The majority of studies are based on in vitro and animal models, which may not fully reflect clinical effects in human populations.
- Variability in bioactive composition: The composition and concentration of bioactive compounds in plant extracts can vary due to multiple factors, including plant sources, environmental conditions, and extraction methods. This variability complicates the establishment of consistent efficacy and safety profiles.
- Confounding variables in study design: Many studies do not adequately control for lifestyle factors such as diet and physical activity. Consequently, observed effects may be influenced by external variables rather than the plant extracts alone.
- Short study durations and small sample sizes: Clinical trials investigating the effects of plant extracts on metabolic syndrome often have limited durations, small sample populations, and insufficient long-term follow-up, restricting the generalizability of findings and their broader clinical application. Large-scale, long-term, randomized controlled trials are essential to establish both efficacy and safety for widespread use.
- Underrepresentation of pediatric and adolescent populations: While metabolic syndrome is predominantly associated with adults and older individuals, its prevalence is increasing among children and adolescents. However, research on the potential benefits of plant extracts in these younger populations remains limited.
- Lack of direct comparisons with conventional therapies: Most clinical studies utilize placebo-controlled designs, with relatively few directly comparing plant extracts to conventional pharmaceutical treatments. Future research should emphasize comparative studies to draw more definitive conclusions regarding the therapeutic potential of plant extracts relative to standard medical interventions.
- Limited investigation into bioavailability: Bioavailability is a crucial determinant of the physiological effects of both pharmacological drugs and plant extracts. However, in contrast to pharmaceutical compounds, the bioavailability of plant-derived extracts in human subjects remains largely unexplored.
- Regulatory and interaction challenges: The absence of standardized regulatory frameworks and the potential for interactions between plant extracts and pharmaceutical drugs present additional challenges that must be addressed to ensure safe and effective use.
- Stability and commercial viability: While the stability and shelf-life of plant extracts have been extensively studied, their evaluation within commercially available products is limited. Further research is needed to optimize formulations for real-world applications.

To fully harness the therapeutic potential of plant extracts, rigorous, well-designed clinical trials are essential to confirm their efficacy, safety, and long-term benefits. Addressing these research gaps will facilitate the integration of plant-based interventions into mainstream healthcare and functional food industries.

## Figures and Tables

**Table 1:** Effects of plant extracts on metabolic syndrome risk factors.

Plant Extract	Study Model	Concentration/Dose	Outcomes	References
*Prunus domestica* L. fruit pulp	In vitro	-	↓ α-amylase, α-glucosidase, HMG-CoA reductase, and pancreatic lipase enzymes ↓ nitrate, PGE_2_, and IL-1β	[33]
*Prunus persica* (L.) Stokes flower	In vivo	0.2% or 0.6% extract mixed with diet for 8 weeks	↓ body weight, visceral fat mass, and serum levels of glucose, ALT, and AST	[34]
*Hibiscus sabdariffa* L., *Vigna unguiculata* L. Walp., and *Solanum nigrum* L. extracts	In vivo	200 mg/kg/day for 12 days	↓ FBG levels	[40]
*Prunus salicina* Lindl. (Queen Garnet plum juice)	Randomized, double-blinded, placebo-controlled, cross-over trial	200 mL/day for 4 weeks	↓ body weight, BMI, leptin levels, and increased adiponectin levels in healthy participants	[41]
Soluble fiber-rich brewer’s spent grain (rich in soluble fibers and ferulic acid)	Randomized, placebo-controlled, double-blind, cross-over clinical trial	4.25 g of extract before OGTT in cross-over design	↓ postprandial glycemia and insulinemia in healthy individuals with slightly impaired glucose tolerance	[42]
Extra-virgin olive oil	Randomized, controlled, cross-over clinical trial	Olive oil consumed as meal	↓ postprandial glycemic response in type 1 diabetic patients	[45]
Olive oil	Interventional study	30 mL/day for 4 weeks	↓ FBG, TC, LDL, and TGs in diabetic patients *↑* HDL	[46]
Olive leaf extract	Randomized, placebo-controlled, clinical trial	500 mg/day for 14 weeks	↓ HbA1c and fasting insulin levels in diabetic patients	[47]
Fenugreek seeds	Randomized, double-blind, placebo-controlled, clinical trial	1 g/day for 2 months	Improvement of glucose and insulin in diabetic patients Non-significant effects on FBG and OGTT	[48]
Fenugreek seeds	Randomized, placebo-controlled, cross-over clinical trial	100 g/day for 10 days	↓ FBG and improvement in OGTT in diabetic patients	[49]
Fenugreek seeds	Interventional study	15 g/day	↓ postprandial glucose levels in diabetic patients	[50]
Green tea extract	Randomized, placebo-controlled, cross-over clinical trial	Packet of green tea extract (containing 544 mg polyphenols) for 2 months	↓ HbA1c levels in healthy subjects No effects on FBG	[51]
Exercise and green tea extract	Randomized, placebo-controlled, cross-over clinical trial	Green tea extract (containing 890 mg polyphenols)	↓ AUC for insulin in healthy subjects ↑ insulin sensitivity	[52]
Epigallo-catechin-3-gallate (800 mg/day)	Randomized, double-blind, clinical trial	Epigallo-catechin-3-gallate (800 mg/day) for 8 weeks	No significant effects on FBG, HbA1c, insulin sensitivity, insulin secretion, and glucose tolerance	[53]
Qinggan Jiangtang tablets	Randomized, controlled, double-blind clinical trial	Three tablets twice a day for 1 month	↓ blood glucose levels, lipid profiles, blood pressure, and insulin resistance in patients with metabolic syndrome	[54]
Grape seed extract (rich in resveratrol and procyanidins)	Randomized, placebo-controlled, clinical trial	100 mg/day for 8 weeks	Improvement of insulin concentration and resistance in adolescents with metabolic syndrome.	[55]
*Zanthoxylum armatum* DC (fruits, leaves, and bark extracts)	In vivo	500 mg/kg/day for 15 days	↓ TC, TG, LGL levels	[61]
Schisandrae Fructus, milk thistle, hawthorn, and bitter melon	In vitro / In vivo	0–1000 µg/mL (in vitro); 2–4 % herbal formula for 12 weeks (in vivo)	↓ adipocyte differentiation, cholesterol uptake, and hepatic lipid accumulation. ↓ body weight, fat pad mass, liver weight, and improved lipid profiles.	[62]
Fixed oils from spices (*Alpinia galanga* (L.) Willd., *Cinnamomum zeylanicum* var. cassia, *Trigonella foenum-graecum* L., *Foeniculum vulgare* Mill., and *Myristica fragrans* Houtt.)	In vitro / In vivo	12.5–100 μg/mL (in vitro); 2.5–12.5% fixed oils mixed with diet (in vivo)	↓ accumulated lipid droplets in 3T3-L1 cell lines. Improvement of lipid profiles and antioxidant enzymes. ↓ droplets in liver and adipose tissues.	[63]
*Mangifera indica* L. leaves extract	In vivo	400 mg/kg for 32 days	↓ TC, TG, LDL, atherogenic index ↑ HDL levels	[64]
Extra-virgin olive oil plus fish oil	Interventional study	Extra-virgin olive oil (10 mL/day) and fish oil (3 g/day) for 3 months	↓ TC, LDL, and oxidative capacity ↑ HDL levels	[65]
*Monascus purpureus*	Randomized, double-blind, placebo-controlled trial	Standardized food supplement (2.8 mg of monacolins) for 3 months	↓ TC and LDL levels ↑ HDL levels	[66]
Prunes	Randomized, placebo-controlled, crossover, clinical trial	100 g for 8 weeks	↓ serum LDL levels and fecal lithocholic acid	[67]
Lettuce seed extract	Randomized, double-blind, placebo-controlled, pilot trial	1000 mg/day for 12 weeks	↓ TC, TG, and LDL levels ↑ HDL levels	[68]
Bergamot extract (120 mg flavonoids), vitamin C, phytosterols, and chlorogenic acid from dry artichoke extract	Randomized, double-blind, placebo-controlled trial	Two pills of food supplement for 8 weeks	↓ TC, TG, LDL, and non-HDL cholesterol levels. ↓ hs-CRP and TNF-α.	[69]
Bitter melon extract	Randomized, placebo-controlled, clinical trial	100 mg for 30 days	↓ LDL levels. No significant effects on TC, TG, and blood glucose levels	[70]
Psyllium fibers	Randomized, placebo-controlled, crossover, clinical trial	6 g/day for 6 weeks	Improvement of fat distribution and lipid profile in healthy adolescents	[71]
Black cherry fruit extract	In vivo	300 mg/kg/day for 4 weeks	↓ oxidative stress markers and systolic blood pressure	[77]
*Adansonia digitata* L.	In vivo	200 mg/kg and 400 mg/kg/day for 3 weeks	Dose-dependent reduction in systolic and diastolic blood pressure, mean arterial pressure, and heart rate. ↓ ACE activity, CRP, IL-1β, malondialdehyde, creatine kinase-MB, and lactate dehydrogenase	[78]
*Prunus persica* (L.) Stokes extract	In vitro	0.5 to 20 μg/mL	Enhanced vasorelaxation by targeting NO-sGC-cGMP and IP3R pathways	[79]
Cruciferous vegetables	Randomized, placebo-controlled, cross-over clinical trial	300 g/day for 2 weeks	↓ systolic blood pressure	[80]
*Nigella sativa* L. seed extract	Randomized, double-blind, placebo-controlled, clinical trial	200 and 400 mg/day for 8 weeks	↓ systolic and diastolic blood pressure	[81]
Garlic extract	Randomized, double-blind, placebo-controlled, clinical trial	960 mg/day (containing 2.4 mg S-allylcysteine) for 12 weeks	↓ systolic blood pressure	[83]
Anthocyanin-rich Queen Garnet plum juice	Randomized, double-blind, placebo-controlled, cross-over trial	200 mL/day for 28 days	↓ ADP-induced platelet aggregation and platelet activation-dependent P-selectin expression. Prolonged activated-partial thromboplastin clotting time. ↓ plasma-fibrinogen and malondialdehyde levels.	[84,85]
*Viburnum trilobum* Marshall, *Amelanchier alnifolia, Shepherdia argentea* (Pursh) Nutt., and *Prunus virginiana* L.	In vitro	-	↓ aldose reductase. ↓ IL-1β and COX-2 gene expression.	[86]

Increase (↑), decrease (↓), prostaglandin E2 (PGE2), interleukin-1β (IL-1β); alanine aminotransferase (ALT), aspartate aminotransferase (AST), fasting blood glucose (FBG), body mass index (BMI), oral glucose tolerance test (OGTT), total cholesterol (TC), low-density lipoprotein (LDL), high-density lipoprotein (HDL), triglycerides (TGs), glycated hemoglobin (HbA1c), area under curve (AUC), high sensitivity C-reactive protein (hs-CRP), tumor necrosis factor-α (TNF-α), angiotensin-converting enzyme (ACE), nitric oxide–soluble guanylate cyclase–cyclic guanosine monophosphate pathway (NO-sGC-cGMP), inositol triphosphate receptors (IP3R), cyclooxygenase (COX-2).

**Table 2:** Encapsulation techniques, their definitions, uses, advantages, and disadvantages [131].

Encapsulation Technique	Definition	Uses	Advantages	Disadvantages
Spray-drying	A method where active ingredients are mixed with a wall material, atomized in a hot chamber, and dried into powder.	Used for shelf-life enhancement and the encapsulation of various active compounds.	Low cost, easy scalability, and improved product stability.	A limited number of wall materials can be used.
Freeze-drying	Freezing active materials to form ice, followed by sublimation in a vacuum to create porous, powdered products.	Encapsulation of temperature-sensitive materials like aromas and volatile oils.	Simple process, preserves sensitive compounds effectively.	Time-consuming and high energy costs.
Extrusion	Polymer solution-containing active material is extruded through a nozzle into a gel solution.	Used for encapsulating both hydrophilic and hydrophobic compounds.	Simple, laboratory-friendly, and produces high shelf-life capsules.	Difficult and expensive to scale up.
Emulsification	Involves creating emulsions of two immiscible liquids (water and oil) stabilized by emulsifiers.	Encapsulation of oil-soluble compounds like dietary fats and sterols.	Provides both liquid and powder encapsulation options.	Requires specific emulsifiers for stabilization.
Coacervation	Separation of phases leading to the formation of encapsulated materials within polymeric walls.	High-efficiency encapsulation with controlled release properties.	High encapsulation efficiency and control over material release.	Capsules are often unstable and the production cost is high.
Molecular inclusion	Based on hydrogen bonding and electrostatic interactions between polar molecules.	Encapsulation of polar molecules, commonly using cyclodextrins.	Compatible with a wide range of polar compounds.	Limited use outside of specific polar interactions.
Ionic gelation	Encapsulation using microbeads in biopolymer gels, formed by methods like spraying or extrusion.	Commonly used for suspending active materials in polymer solutions.	Simple and adaptable to various active materials.	Limited by the biopolymer’s properties and stability in different environments.

## Abbreviations

ADRs	adverse drug reactions
ALT	alanine aminotransferase
AST	aspartate aminotransferase
BMI	body mass index
CHD	coronary heart disease
DSHEA	Dietary Supplement Health and Education Act
EMA	European Medicines Agency
FDA	Food and Drug Administration
HbA1c	glycated hemoglobin
HDL	high-density lipoprotein
HPLC	high-performance liquid chromatography
hs-CRP	high sensitivity C-reactive protein
IDF	International Diabetes Federation
LDL	low-density lipoprotein
MAE	microwave-assisted extraction
NCEP ATP III	National Cholesterol Education Program’s Adult Treatment Panel III
NO-cGMP	nitric oxide–cyclic guanosine monophosphate pathway
NO-sGC-cGMP	nitric oxide–soluble guanylate cyclase–cyclic guanosine monophosphate pathway
PLE	pressurized liquid extraction
ROS	reactive oxygen species
SFE	supercritical fluid extraction
T2DM	type 2 diabetes mellitus
TC	total cholesterol
TGs	triglycerides
UAE	ultrasound-assisted extraction
VLDL	very-low-density lipoprotein
WHO	World Health Organization
